# Molecular characterization of three common olive (*Olea europaea* L.) cultivars in Palestine, using simple sequence repeat (SSR) markers

**DOI:** 10.1080/13102818.2014.957026

**Published:** 2014-10-28

**Authors:** Ramiz Obaid, Hassan Abu-Qaoud, Rami Arafeh

**Affiliations:** ^a^ Palestinian Ministry of Agriculture, Olive Department, Qalqilia District, Palestine; ^b^ Department of Plant Production and Protection, Faculty of Agriculture, An-Najah National University, Nablus, Palestine; ^c^ Biotechnology Research Center, Palestine Polytechnic University, Hebron, Palestine

**Keywords:** microsatellite variation, olive, *Olea europaea* L, genetic polymorphism

## Abstract

Eight accessions of olive trees from three common varieties in Palestine, Nabali Baladi, Nabali Mohassan and Surri, were genetically evaluated using five simple sequence repeat (SSR) markers. A total of 17 alleles from 5 loci were observed in which 15 (88.2%) were polymorphic and 2 (11.8%) were monomorphic. An average of 3.4 alleles per locus was found ranging from 2.0 alleles with the primers GAPU-103 and DCA-9 to 5.0 alleles with U9932 and DCA-16. The smallest amplicon size observed was 50 bp with the primer DCA-16, whereas the largest one (450 bp) with the primer U9932. Cluster analysis with the unweighted pair group method with arithmetic average (UPGMA) showed three clusters: a cluster with four accessions from the ‘Nabali Baladi’ cultivar, another cluster with three accessions that represents the ‘Nabali Mohassen’ cultivar and finally the ‘Surri’ cultivar. The similarity coefficient for the eight olive tree samples ranged from a maximum of 100% between two accessions from Nabali Baladi and also in two other samples from Nabali Mohassan, to a minimum similarity coefficient (0.315) between the Surri and two Nabali Baladi accessions. The results in this investigation clearly highlight the genetic dissimilarity between the three main olive cultivars that have been misidentified and mixed up in the past, based on conventional morphological characters.

## Introduction

Olive tree (*Olea europaea* L.; *Oleaceae*) is one of the most globally important long-lived Mediterranean fruit trees. The number of olive tree cultivars in the world exceeds 1200; about 1200 named olive tree varieties with over 3000 synonyms have been ascertained.[1–3] There is much confusion and uncertainty concerning the identity of many olive tree varieties.[4,5] Different techniques have been adopted to evaluate olive diversity; primarily, morphological[6] and biological characters that have been widely applied for descriptive purposes and are commonly used to distinguish olive tree cultivars.[1,7–14] Several molecular markers have been recently used to characterize and discriminate olive tree cultivars, such as chloroplast DNA restriction fragment length polymorphism (RFLP),[15] chloroplast DNA simple sequence repeats (SSRs),[4,16] amplified fragment length polymorphism (AFLP),[12,17–20] random amplified polymorphic DNA (RAPD),[14,16,21–25] mitochondrial DNA RFLP,[15,26] isozymes,[27] inter simple sequence repeat (ISSR) molecular markers,[28] a combination of ISSR and SSR,[29] SSR and RAPD,[24] SSR [16,30–40] and plastome sequence comparison.[41] In the Euro-African Mediterranean region, many studies on olive tree polymorphism have been undertaken [42]; for example, intra-varietal genetic variability among 120 clones of the Portuguese olive ‘Cobrançosa’ cultivar was investigated using RAPD and ISSR techniques.[11] Another study on Italian cultivars was conducted using AFLP analysis, and significant genetic diversity was revealed.[43] RAPD analysis of 84 olive accessions in Tunisia indicated the coefficient of similarity ranges from 0.98 to 0.40, estimated by a simple matching algorithm.[44] RAPD, AFLP and SSR markers were compared for the identification and genetic differentiation of 32 Spanish and Italian olive tree cultivars. Based on SSR co-dominancy, a high level of polymorphism and discrimination power was reported, as well as ideal olive genome mapping and genetic studies were presented.[45]

In Palestine, there are many olive tree cultivars; however, among them the most common ones are ‘Nabali Baladi’ (NB), ‘Nabali Mohassan’ (NM) and ‘Surri’. To the best of our knowledge, there are few reports on the morphological, phenological, bio-agronomical and productive characteristics of these cultivars to date. Despite the socio-economical importance of olive tree cultivation and the health benefits of olive-derived products, studies addressing olive tree genetic diversity in this region remain insufficient. Therefore, this study was conducted to genetically discriminate between the major Palestinian olive tree cultivars, using microsatellite (SSR) markers.

## Materials and methods

### Plant material

Eight olive trees were chosen after accurate field observations in Qalqilia District (north-west of West Bank) as a representative sample of true varieties. To exclude any developmental variation among the three cultivars, leaf samples were taken from 30-year-old fruiting trees. The three analysed varieties are NB, NM and Surri, represented by four, three and one accession, respectively.

### DNA isolation and purification

About 100 mg of silica dried leaf tissue was used in the total genomic DNA isolation and purification. DNA extraction was carried out with DNeasy Plant Mini-prep Kit from QIAGEN (QIAGEN, Valencia, CA), according to the manufacturer's instructions. DNA was suspended in Tris-EDTA (TE) buffer; then, each sample was diluted up to 60 ng/μL before conducting a polymerase chain reaction (PCR).

### Microsatellite analysis

A total of five microsatellite primer pairs were used to test the polymorphism in the eight olive accessions. The primers were selected from previous literature: DCA9, DCA16,[46,47] GAPU103,[8] UDO99-28 and UDO99-39,[48] and were chosen for their high discriminative power. The procedure for SSR amplification was carried out as described by Muzzalupo et al.[35] A list of microsatellite primers along with forward and reverse sequences, used to survey polymorphism, is given in Table 1.

**Table 1.** List of SSR primer pairs used in this study, along with their forward and reverse sequences.

No.	SSR primer code	Primers sequence (forward – reverse)
1	U9935	F: 3′ AATTTAATGGTCACACACAC 5′ R: 3′ ATTGCGAAATAGATCTACGA 5′
2	U9928	F: 3′ CTGCAGCTTCTGCCCATAC 5′ R: 3′ GCAGCTCATCATTTGGCACT 5′
3	GAPu103	F: 3′ TGAATTTAACTTTAAACCCACACA 5′ R: 3′ GCATCGCTCGATTTTATCC 5′
4	DCA9	F: 3′ AATCAAAGTCTTCCTTCTCATTTCG 5′ R: 3′ GATCCTTCCAAAAGTATAACCTCTC 5′
5	DCA16	F: 3′ TTAGGTGGGATTCTGTAGATGGTTG 5′ R: 3′TTTTAGGTGAGTTCATAGAATTAGC 5′

### Polymerase chain reaction

PCR amplication was carried out in a total volume of 25.0 μl containing 1.0 μl of genomic DNA template (30–60 ng), 22.0 μL of Master Mix which contains (15.5 μL H_2_O, 2.5 μL of 10X buffer containing (75.0 mmol/L Tris-HCl, 20 mmol/L (NH_4_)_2_SO_4_, 3.0 mmol/L MgCl_2_ and 0.01% (V/V) Tween® 20), l 2.5 μL of MgCl_2_, 0.2 mmol/L of each of deoxiadenosine triphosphate (dATP), deoxicytidine triphosphate (dCTP), deoxiguanosine triphosphate (dGTP) and deoxithymidine triphosphate (dTTP), respectively, and 5 units (0.2 μL) of Taq polymerase enzyme (New England Biolabs).

Forward and reverse primers were added at 1.0 μL each (15 pmol/μL). The PCR reactions were set up in 0.2 mL PCR tubes. PCR reactions were carried out in an Applied Biosystems thermal cycler. The PCR was programmed for all tested primers at 5 min initial denaturation step at 94 °C, followed by 35 cycles at 95 °C (1 min), annealing at 55 °C (1 min) and extension at 72 °C (2 min). The amplification cycles were immediately followed by an additional extension step for 7 min, and then finally samples were held at 4 °C. PCR products were then loaded onto 2.0% (w/v) agarose gels containing ethidium bromide and electrophoresed at 100 V for 1.5 h. Following electrophoresis, the gels were photographed with an ordinary gel documentation system with a UV screen.

### Scoring of SSR bands and data analyses

The DNA bands were scored as (1) for the bands present and (0) for the bands absent. Based on the banding pattern scored, a similarity matrix among olive tree accessions was calculated using SIMQUAL (Similarity of Qualitative Data). Cluster analysis was performed on the estimated similarities, using the unweighted pair group method with arithmetic average (UPGMA) and SHAN algorithm, and the resulting clusters were expressed as a dendrogram, using NTSYS-PC (Exeter Software v.2.02).

Per cent polymorphic loci were calculated using the following formula: *P*_s_ = number of polymorphic loci/total number of loci. The similarity matrix was calculated using the formula of Dice's coefficient [28]: Dice = 2*a*/(2*a* + *b* + *c*).

## Results and discussion

**Figure 1.**
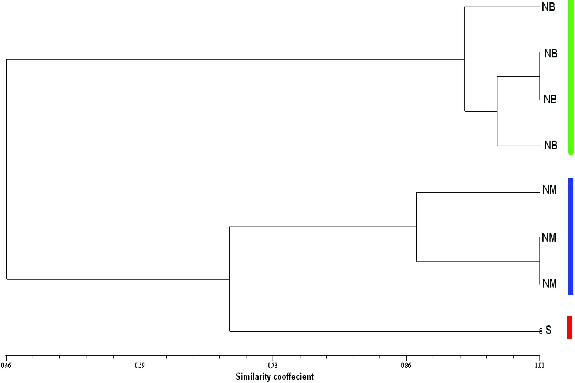
UPGMA dendrogram of 8 olive accessions based on Dice's similarity coefficients, using 17 SSR markers. NB: Nabali Baladi; NM: Nabali Mohassan; S: Surri.

A total of 17 alleles over 5 loci were observed with 15 polymorphic (88.2%) and 2 monomorphic (11.7%) amplicons. This is comparable to the number of alleles revealed among olive tree cultivars reported by Muzzalupo et al. [35] and Cipriani et al. [48] but somewhat lower than that published by Lopes et al.,[49] Sarri et al. [50] and Soleimani et al.,[25] probably because their studies involved a large number of cultivars. An average of 3.4 alleles per locus was reported, ranging from 2.0 with the primer pair GAPU-103 and DCA9 to 5.0 with U99-32 and DCA16. The smallest allele size was 50 bp among the five loci and was observed with the primer pair DCA16, whereas the largest allele size was 450 bp observed with U99-32. The clustering pattern in the UPGMA dendrogram (Figure 1) revealed three groups, in which NB appeared in one cluster, NM grouped in the second cluster and the last accession, Surri, appeared as a separate Operational Taxonomic Unit (OTU). Furthermore, two of the NM accessions and two of the NB accessions were found to have 100% genetic similarity. The lowest genetic similarity was revealed between Surri and NB samples (32%). The similarity range was comparable to the result in the report of Muzzalupo et al.[35] When compared with the above-reported findings, the polymorphism ratio observed among the three olive tree cultivars investigated here is close to what other researchers [22,46,49] have reported.

The characterization of the morphological and physiological cultivar traits is based on specific plant developmental stages and ontogeny,[51] related to the performance of a specific plant, such as its ability to resist stress conditions.[52] In this regard, we excluded any possible developmental variation among the three cultivars by taking homogeneous leaf samples from trees of similar age with specific morphological and physiological appearance. Unlike the Surri variety, which has asymmetric and violet ripe fruits, the NB and NM varieties differ in specific characters, such as leaf shape, oil properties and fatty-acid composition [53] and different sensitivity to pests.[54–56] Since the two varieties have a similar Arabic name, they are more often than not confused. One important achievement in this study is the discrimination between these two varieties with the sensitive SSR molecular marker, so a genetic barcode is now established. One of the benefits could be that the agricultural authorities would have better opportunities in issuing reliable certificates for proper identity of the propagated material.

One of the main findings in this study is the validation of morphological characters previously analysed [57] in these three varieties for cultivar discrimination and the clarification of local cultivars’ identity and their relationships within this part of the eastern Mediterranean. The use of SSR markers led to very interesting findings. When molecular data were compared in order to detect the level of reliability for the morphological parameters and to provide information on which parameters should be useful to discriminate olive tree cultivars, no ambiguous cases of synonymy were found. This means that all of the cultivars examined were different from each other. The cultivars were able to be distinguished even when they originated from the same area. On the other hand, the marked genetic variability observed among the eight samples indicated a situation of ‘cultivar populations’, that is, the presence of different clones within the same cultivar. The results of this study provide important information about Palestinian olive germplasm, for which, until now, only few studies on a very limited number of samples have been carried out.

## Conclusions

In this study, SSR was found to be useful for detecting genetic differences among Palestinian major olive cultivars. The result of this study provides vital information about Palestinian olive germplasm; it may also help breeders on selecting the most diverse genotypes with similar fruit characteristics to begin breeding programmes. This may improve olive growing and production.
